# A novel alternative for pyrogen detection based on a transgenic cell line

**DOI:** 10.1038/s41392-024-01744-0

**Published:** 2024-02-19

**Authors:** Qing He, Chuan-Fei Yu, Gang Wu, Kai-Qin Wang, Yong-Bo Ni, Xiao Guo, Zhi-Hao Fu, Lan Wang, De-Jiang Tan, Hua Gao, Can Wang, Gang Chen, Xu-Hong Chen, Bo Chen, Jun-Zhi Wang

**Affiliations:** 1 National Institutes for Food and Drug Control, Beijing, China; 2 School of Life Science and Biopharmaceutics, Shenyang Pharmaceutical University, Shenyang, China; 3 Shanghai Institute for Food and Drug Control, Shanghai, China; 4 KeyMed Biosciences Inc., Chengdu, China

**Keywords:** Biological models, Predictive markers

## Abstract

Pyrogen, often as a contaminant, is a key indicator affecting the safety of almost all parenteral drugs (including biologicals, chemicals, traditional Chinese medicines and medical devices). It has become a goal to completely replace the in vivo rabbit pyrogen test by using the in vitro pyrogen test based on the promoted ‘reduction, replacement and refinement’ principle, which has been highly considered by regulatory agencies from different countries. We used NF-κB, a central signalling molecule mediating inflammatory responses, as a pyrogenic marker and the monocyte line THP-1 transfected with a luciferase reporter gene regulated by NF-κB as an in vitro model to detect pyrogens by measuring the intensity of a fluorescence signal. Here, we show that this test can quantitatively and sensitively detect endotoxin (lipopolysaccharide from different strains) and nonendotoxin (lipoteichoic acid, zymosan, peptidoglycan, lectin and glucan), has good stability in terms of NF-κB activity and cell phenotypes at 39 cell passages and can be applied to detect pyrogens in biologicals (group A & C meningococcal polysaccharide vaccine; basiliximab; rabies vaccine (Vero cells) for human use, freeze-dried; Japanese encephalitis vaccine (Vero cells), inactivated; insulin aspart injection; human albumin; recombinant human erythropoietin injection (CHO Cell)). The within-laboratory reproducibility of the test in three independent laboratories was 85%, 80% and 80% and the interlaboratory reproducibility among laboratories was 83.3%, 95.6% and 86.7%. The sensitivity (true positive rate) and specificity (true negative rate) of the test were 89.9% and 90.9%, respectively. In summary, the test provides a novel alternative for pyrogen detection.

## Introduction

Pyrogens, including endotoxins and nonendotoxins, can often be present as contaminants in parenteral drugs and can cause severe, even life-threatening, fevers in patients.^1,2^ Pyrogen testing is a statutory requirement listed in pharmacopoeias for controlling the safety of parenteral drugs and research on feasible pyrogen tests is urgently needed.^3,4^

The developed pyrogen detection tests ranged from the rabbit pyrogen test (RPT) to the bacterial endotoxin test (BET), followed by the monocyte activation test (MAT).^5,6^ The RPT and BET, as traditional pyrogen tests, have been adopted in pharmacopoeias and widely used to date, but both have flaws. RPT is an in vivo test, in addition to its poor reproducibility and high cost.^7,8^ The BET, based on the combination of a bacterial endotoxin with limulus amoebocyte lysate to form a gel, can detect only gram-negative bacterial endotoxins.^9,10^ As a core component of the limulus amoebocyte lysis reagent, recombinant factor C can replace this reagent and be used to specifically detect bacterial endotoxins.^11–13^ In view of the above problems, the overall trend in the field of research and regulation has been to establish MATs.^14–16^ MATs use human immune cells [including peripheral blood mononuclear cells (PBMCs),^17–19^ whole-blood (WB) cells^20–22^ and MM6 cells^23,24^] that are incubated with drugs and pyrogens as an in vitro model and use proinflammatory cytokines (including IL-6 and IL-1β) secreted by these cells as pyrogenic indicators. However, MATs based on human blood often need much blood and exhibit variations across individuals, so their convenience and standardisation need to be improved to increase the use of MATs. Moreover, it is difficult to obtain monocytic cell lines (MM6—IL-6 test) due to patents.

Fever is essentially the systemic inflammatory response induced by pyrogens. Exogenous pyrogens, as pathogen-associated microbial patterns (PAMPs), activate immune cells (including monocytes and macrophages) by recognising pattern recognition receptors (PRRs) and inducing those cells to secrete a series of proinflammatory factors (also termed endogenous pyrogens), which act on the thermoregulation centre of the human brain, causing fever in turn.^25^ There is a certain rationality to use proinflammatory cytokines as pyrogenic markers; however, pyrogens stimulate immune cells via different signalling pathways to secrete different proinflammatory factors. For example, the lipopolysaccharide (LPS) of gram-negative bacteria^18^ binds to Toll-like receptor (TLR) 4 and activated TLR4 can induce proinflammatory responses through MyD88-dependent and TRIF-dependent pathways, both of which include the activation of NF-κB.^26,27^ Lipoteichoic acid (LTA) of gram-positive bacteria mainly binds TLR2 and then activates NF-κB to induce the production of proinflammatory mediators (TNF-α, IL-1, IL-6, IL-8 and NO).^28,29^ Zymosan produced by fungi can activate NF-κB by binding TLR2 to induce the expression of proinflammatory factors (TNF-α, IL-1β and IL-8).^30,31^ The activation of NF-κB is involved in the mechanism by which most pyrogens stimulate the secretion of proinflammatory factors in humans,^32^ and it may be reasonable to use NF-κB as a representative pyrogenic marker (Supplementary Fig. S1a).

Based on the above proposal, we constructed the transgenic human monocyte line THP-1 stably transfected with a luciferase reporter gene vector containing the DNA response element of NF-κB (Supplementary Fig. S1b) to show that the proposed test could be a novel alternative for pyrogen detection.

## Results

### Establishing the THP-1_NF-κB_luc_cell subclone that is sensitive to endotoxin

**Fig. 1:**
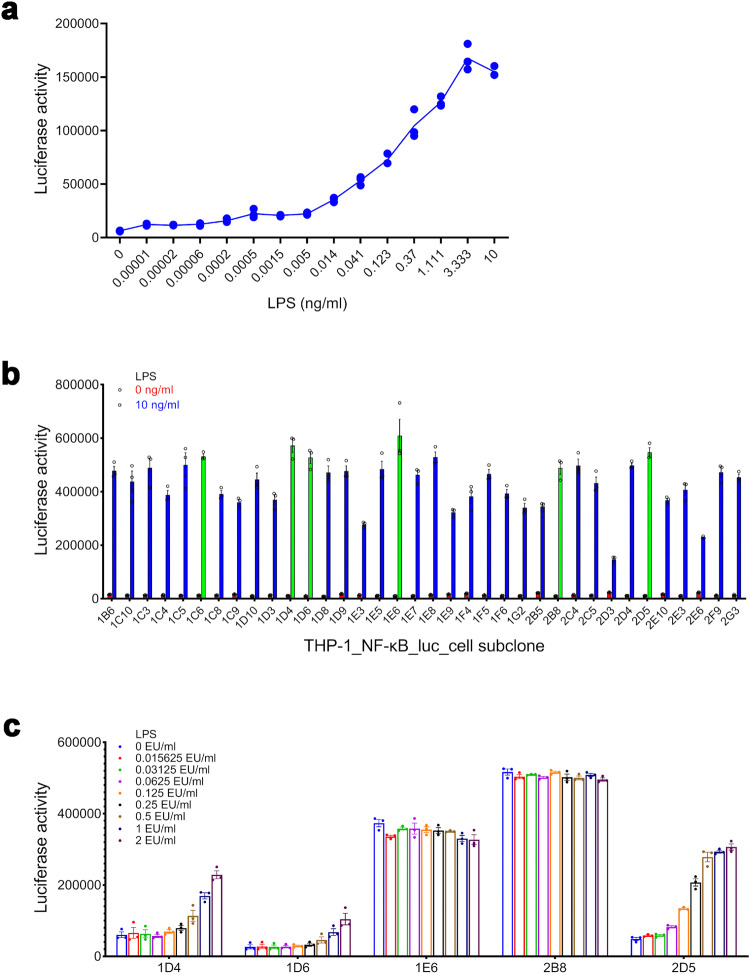
Establishing the THP-1_NF-κB_luc_cell subclone that is sensitive to endotoxin. The NF-κB response of the THP-1_NF-κB_luc_cell mix pool (**a**) at a density of 4 × 10^4^ cells/ml stimulated by LPS (from *Escherichia coli* O127:B8) for 24 h (*n* = 3). **b** The NF-κB response of the THP-1_NF-κB_luc_cells subclone at a density of 4 × 10^4^ cells/ml stimulated by LPS (from *Escherichia coli* O127:B8) for 24 h (*n* = 3). The NF-κB response of the THP-1_NF-κB_luc_cell subclone (**c**) at a density of 4 × 10^4^ cells/ml stimulated by LPS (the national standard for bacterial endotoxins) for 20 h (*n* = 3)

Both RPT and MAT are not convenient.^33,34^ To establish an in vitro model that is easy to use, we constructed a transgenic cell line by transfecting a luciferase reporter gene regulated by NF-κB into the human monocyte line THP-1,^35,36^ which was screened for the NF-κB response by treatment with serial concentrations of LPS. The suitable subclone was selected mainly based on the signal-to-noise ratio (SNR) of the subclone’s NF-κB response, which was calculated as the subclone’s NF-κB response to LPS divided by that of the negative control. If the background (negative control) of the NF-κB response is too high, it will alter the detection limit when testing LPS. Based on this consideration, the principles of selecting a suitable subclone were as follows: (1) high SNR; (2) low background; and (3) good cell growth. LPS activated the NF-κB response of the THP-1_NF-κB_luc_cell mix pool in a dose-dependent manner (Fig. 1a). Six subclones (including 1C6, 1D4, 1D6, 1E6, 2B8 and 2D5) with relatively high NF-κB activity against LPS were screened from the 36-well-grown subclones (Fig. 1b). The NF-κB activity of subclone 2D5 had a relatively good dose-dependent response to LPS (Fig. 1c). The background (negative control) of the NF-κB response of subclone 1C6 was too high; thus, the NF-κB response of subclone 1C6 had no dose-dependent relationship with the concentration of LPS (data not shown), which was similar to that of subclone 2B8 (Fig. 1c). Therefore, the subsequent experiments did not include subclone 1C6. Based on the results, subclone 2D5 was selected to establish the test.

### Confirming the experimental parameters (the cell density, the pyrogen stimulation duration) of the test and the NF-κB response of THP-1 cells to endotoxin and nonendotoxin pyrogens

**Fig. 2:**
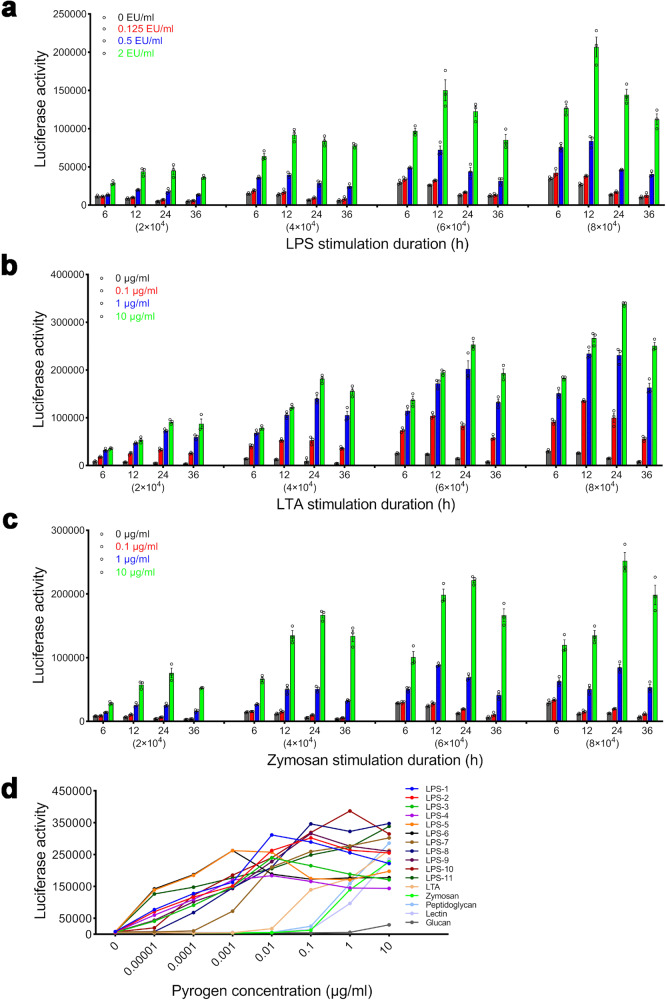
Confirmation of the experimental parameters of the test and the NF-κB response of THP-1 cells to endotoxin and nonendotoxin pyrogens. NF-κB response of THP-1 cells at different densities (2 × 10^4^, 4 × 10^4^, 6 × 10^4^, 8 × 10^4^ cells/well) after stimulation with endotoxin (LPS (the national standard for bacterial endotoxins) from gram-negative bacteria (**a**)) and nonendotoxin (LTA from gram-positive bacteria (**b**) zymosan from fungi (**c**)) for different times (6, 12, 24 and 36 h) (*n* = 3). **d** The dose-effect relationships of pyrogens (e.g., LPS from different strains and nonendotoxin pyrogens, including peptidoglycan, lectin, and glucan) in activating NF-κB in THP-1 cells at a density of 4 × 10^4^ cells/well after stimulation for 24 h (*n* = 4). LPS-1: from *Escherichia coli* O26:B6; LPS-2: from *Escherichia coli* O55:B5; LPS-3: from *Escherichia coli* O111:B4; LPS-4: from *Escherichia coli* O127:B8; LPS-5: from *Escherichia coli* O128:B12; LPS-6: from *Salmonella enterica* serotype Abortus equi; LPS-7: from *Salmonella enterica* serotype enteritidis; LPS-8: from *Salmonella enterica* serotype Minnesota; LPS-9: from *Salmonella enterica* serotype typhimurium; LPS-10: from *Salmonella typhosa*; LPS-11: from *Klebsiella pneumoniae*; Lectin: from *Phaseolus vulgaris* (red kidney bean); Peptidoglycan: from *Bacillus subtilis*; β-1,3-glucan: from *Euglena gracilis*

The BET can only detect gram-negative bacterial endotoxins.^9,10^ To show that the test can detect both endotoxins and nonendotoxins, we studied the dose-effect relationships between 11 kinds of LPSs from different strains and 5 kinds of nonendotoxin pyrogens and the NF-κB response after confirming the suitable experimental parameters of the test. With the extension of stimulation duration, the absolute NF-κB response of THP-1 cells at different densities to pyrogens (LPS (Fig. 2a), LTA (Fig. 2b) and zymosan (Fig. 2c)) had a maximum at 12–24 h and the background (negative control) of the NF-κB response decreased; however, with increasing cell density, the background (negative control) of the NF-κB response increased. At 24 h, the NF-κB response of THP-1 cells at different densities to a low concentration of LPS (0.125 EU/ml) was significantly higher than that of the negative control (*P* < 0.05), and no significant difference in the response was detected at other time points (6, 12 and 36 h), with the exception that the responses at the densities of 6 × 10^4^ and 8 × 10^4^ were significantly higher than that of the negative control (*P* = 0.001, *P* = 0.002) at 12 h. At 24 h, the relative NF-κB response of THP-1 cells at a density of 4 × 10^4^ cells/well to the high concentration of pyrogens (8 EU/ml LPS, 100 μg/ml LTA/zymosan) compared to that of the negative control had the maximum (data not shown). Based on the results, the cell density and the stimulation duration adopted for the test were 4 × 10^4^ cells/well and 24 h, respectively.

There were also significant dose-effect relationships between LPS from different strains, including *Escherichia coli* O26:B6, *Escherichia coli* O55:B5, *Escherichia coli* O111:B4, *Escherichia coli* O127:B8, *Escherichia coli* O128:B12, *Salmonella enterica* serotype Abortus equi, *Salmonella enterica* serotype enteritidis, *Salmonella enterica* serotype Minnesota, *Salmonella enterica* serotype typhimurium, *Klebsiella pneumoniae* and other nonendotoxin pyrogens, including peptidoglycan, lectin, glucan and the NF-κB response (Fig. 2d). The coefficients of variation (CVs) of the response were in the range of 4–25%. High concentrations of LPS (100 μg/ml LPS-1 from *Escherichia coli* O26:B6, 100 μg/ml LPS-4 from *Escherichia coli* O127:B8 and 100 μg/ml LPS-5 from *Escherichia coli* O128:B12) reduced the viability of THP-1_NF-κB_luc_cells, and the concentration range of pyrogens shown in Fig. 2d did not reduce the viability of THP-1_NF-κB_luc_cells. Meanwhile, the test used the national standard for bacterial endotoxin to evaluate the pyrogen contamination of the tested samples and the concentration range of the national standard for bacterial endotoxin used in the test did not reduce the viability of THP-1_NF-κB_luc_cells (data not shown).

### Stability of the cell reactivity and cell phenotypes at different cell passages

**Fig. 3:**
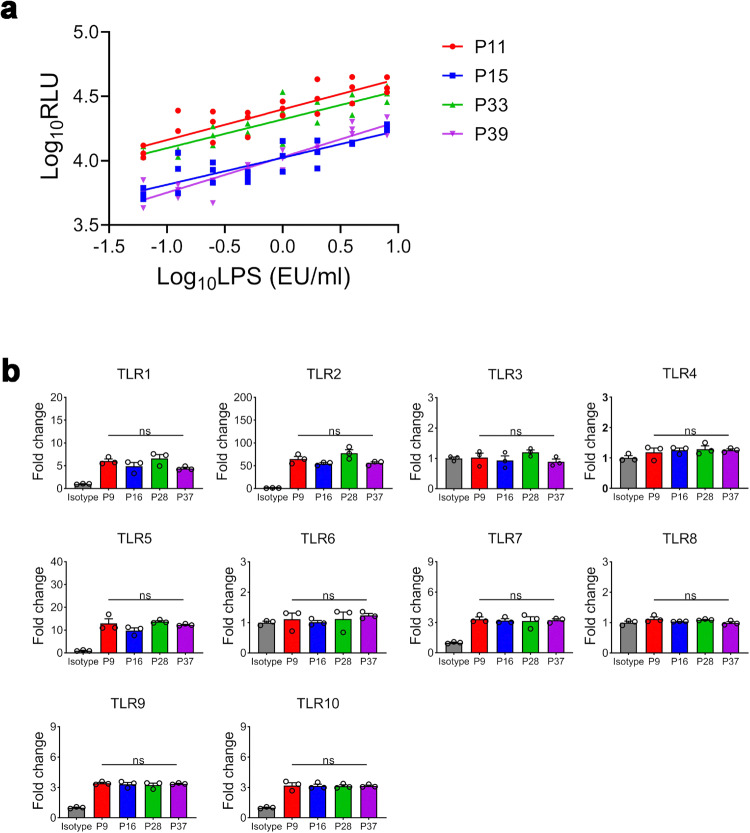
Stability of cell reactivity and cell phenotypes at different cell passages. **a** Dose-effect relationships of LPS in activating NF-κB in THP-1 cells at passages 11, 15, 33 and 39 with a density of 4 × 10^4^ cells/well after stimulation for 24 h (*n* = 3). **b** Expression of TLR1-10 by the transfected THP-1 cells at P6, P16, P28 and P37 was analysed by FACS. Mouse IgG1 was used as an isotype control

Both RPT and MAT have different degrees of variation.^8,22^ To demonstrate the stability of the test, the dose-effect responses and the cell phenotypes at different cell passages were examined. LPS had stable dose-dependent relationships with the NF-κB activity of THP-1 cells at passages (P)11, P15, P33 and P39 and the limit of detection (LOD) of the LPS test was 0.0625 EU/ml (*P* = 0.005 vs. the negative control) (Fig. 3a). The CVs of RLUs were less than 20%. The expression of TLR1-10 by the transfected THP-1 cells at P6, P16, P28 and P37 had no significant change (Fig. 3b). The results suggest that the cells had stable reactivity and phenotypes at different cell passages.

### Precision and accuracy of the established test to quantitatively detect endotoxin (LPS) samples in the laboratory^33^

Four concentrations of LPS samples, with expected concentrations of 0.125, 0.5, 2.0 and 8.0 EU/ml, were detected by three independent runs of the test. The intraassay and interassay CVs of the measured concentrations ranged from 7–21 and 2–16%, respectively (Table 1). A linear relationship was confirmed between the expected (*x*) and measured (*y*) concentrations, *y* = 1.0318x − 0.0679 (*R*^2^ = 0.9986), in the LPS concentration range of 0.125–8.0 EU/ml. The results suggest that the test has good precision and accuracy in the laboratory.

**Table 1:** Precision of the test for detecting LPS in the laboratory (mean ± SD, *n* = 4)

Expected concentration (EU/ml)	Measured concentration (EU/ml)			Interassay CV (%)
	Assay 1	Assay 2	Assay 3	
0.125	0.219 ± 0.027	0.206 ± 0.044	0.159 ± 0.029	16
Intraassay CV (%)	12	21	18	
0.5	0.462 ± 0.066	0.481 ± 0.070	0.461 ± 0.082	2
Intraassay CV (%)	14	15	18	
2	1.790 ± 0.127	1.706 ± 0.203	1.892 ± 0.379	5
Intraassay CV (%)	7	12	20	
8	8.802 ± 0.864	7.948 ± 0.521	7.948 ± 0.521	6
Intraassay CV (%)	10	12	20	

### Applicability of the test with different classes of biologicals^33^

To demonstrate that the test can be used to detect pyrogen in different products, an LPS recovery assay was performed to exclude interference by the drugs themselves on the activation of NF-κB. Drugs within the maximum valid dilution (MVD) do not interfere when the recovery of LPS spikes in drugs is within the range of 50–200%. The MVD is the endotoxin limit concentration of a drug in EU/ml/the LOD of LPS used in the experiment (in this case 0.5 EU/ml). The spike recovery is 100% [the mean concentration of LPS detected in the diluted solution containing the added LPS spike - that detected in the diluted solution]/the added LPS spike (in this case, 1.0 EU/ml). The results (Table 2) suggest that the test has the potential to be widely applied.

**Table 2:** Recovery of LPS spikes in biologicals

Drug		NF-κB response	
	Fold-dilution	Spike recovery (%)	Interference
Group A & C meningococcal polysaccharide vaccine	2000	61	no
Basiliximab	16	75	no
Rabies vaccine (Vero cells) for human use, freeze-dried	100	119	no
Japanese encephalitis vaccine (Vero cells), inactivated	100	111	no
Insulin aspart injection	160	63	no
Human albumin	2	79	no
Recombinant human erythropoietin injection (CHO Cell)	24	56	no

### Validation of the test in different laboratories^15,16,33^

Three independent laboratories participated in the validation of the test. Four drugs (Table 3) were tested at their MVD, each of which was calculated as the endotoxin limit concentration in EU/ml divided by the LOD of LPS used in the experiment (in this case 0.5 EU/ml). Each drug at its MVD was designed to present five blinded spikes (0, 0.25, 0.5, 0.5 and 1.0 EU/ml), two of which were below 0.5 EU/ml (0 and 0.25 EU/ml) and were defined as nonpyrogenic, while three were pyrogenic (0.5, 0.5 and 1.0 EU/ml). Each of the laboratories tested those blinded samples via three independent runs.

**Table 3:** Drugs involved in the validation experiment

Drug	Endotoxin limit concentration (EU/ml)	Limit of endotoxin detection (EU/ml)	Maximum valid dilution
Group A & C meningococcal polysaccharide vaccine	1000	0.5	2000
Basiliximab	8	0.5	16
Rabies vaccine (Vero cells) for human use, freeze-dried	50	0.5	100
Recombinant human erythropoietin injection (CHO Cell)	12	0.5	24

A prediction model was designed to classify the samples after showing interference with the activation of NF-κB. Using this model, the samples were classified as negative when the mean concentration of endotoxin equivalents in each of the sample replicates calculated by the endotoxin standard curve was less than the endotoxin limit concentration specified for the samples. Otherwise, the samples were classified as positive.

Within-laboratory reproducibility was calculated as the proportion of samples classified identically in three independent runs. Interlaboratory reproducibility was calculated as the proportion of samples classified identically in each pairwise laboratory comparison.

The within-laboratory reproducibility of the test in three independent laboratories was 85%, 80% and 80%, and the interlaboratory reproducibility among laboratories was 83.3%, 95.6% and 86.7%, respectively; the sensitivity (true positive rate) and specificity (true negative rate) of the test were 89.9% and 90.9%, respectively (Table 4). The results suggest that the test has good stability and accuracy in different laboratories.

**Table 4:** Validation of the test in different laboratories

Test	Within-laboratory reproducibility (%)	Interlaboratory reproducibility (%)	Sensitivity (%)	Specificity (%)
THP-1/NF-κB	Lab. 1: 85 (51/60)	Lab. 1—Lab. 2: 83.3 (50/60)	89.9 (89/99)	90.9 (60/66)
	Lab. 2: 80 (48/60)	Lab. 1—Lab. 3: 95.6 (43/45)		
	Lab. 3: 80 (36/45)	Lab. 2—Lab. 3: 86.7 (39/45)		
Rabbit pyrogen test^19^	/	/	57.9	88.3

Lab. 1: National Institutes for Food and Drug Control; Lab. 2: Shanghai Institute for Food and Drug Control; Lab. 3: KeyMed Biosciences Inc

## Discussion

RPT cannot truly reflect the human response to pyrogens and requires considerable manpower and materials, leading to a high cost, so it is gradually becoming obsolete.^37–39^ BET is simple and economical. However, BET can only detect bacterial endotoxins from gram-negative bacteria,^40^ which is often interfered with when used to test solutions with a high protein content.^9,10^ Horseshoe crabs have been protected by the state, leading to the suspended production of some Tachypleus amebocyte lysate reagents. Currently, the convenience and ability to standardise MATs based on the use of human WB and PBMCs are key factors that limit the use of those tests. For example, based on our experience, it takes approximately 2 h to prepare fresh PBMCs from a donor and it takes 3–4 h to perform the PBMC test by one experimenter to prepare both PBMCs and tested samples. At the same time, due to the differences in pyrogen reaction between different individuals, the standard use of human blood is a key point to control the stability of this method but is also a major challenge for the standardisation of the test. For the shortcomings of RPT, BET and MATs, as shown in the study, the test was an in vitro pyrogen detection test and it took only 1.5–2 h to operate the test by one experimenter to prepare the cells and tested samples, which was easy to perform and standardize. Meanwhile, the transfected THP-1 cells could detect endotoxin and nonendotoxin pyrogens and exhibited relatively stable expression of various TLRs (TLR1-TLR10) that were the most important PRRs involved in innate immune responses by binding exogenous pyrogens,^41^ which was one of the main factors determining whether the transgenic cells could stably detect those pyrogens.

To demonstrate that the test could overcome the challenges in pyrogen detection due to the products’ complexity of composition and the diversity of processes, we selected a variety of representative biological products (group A and C meningococcal polysaccharide vaccine, inactivated vaccines (rabies vaccine (Vero cells) for human use, freeze-dried; Japanese encephalitis vaccine (Vero cells), inactivated), human albumin, reconstituted product (recombinant human erythropoietin injection (CHO Cell)) and monoclonal antibody (basiliximab)) and the recovery of LPS spikes in biologicals indicated that this method had potential wide applicability. Furthermore, the test had good precision, accuracy, reproducibility, sensitivity and specificity, of which the stability and predictability were comparable to current MATs (fresh or cryopreserved WB—IL-6/IL-1β, MM6—IL-6 tests). The within-laboratory/interlaboratory reproducibility and sensitivity (true positive rate)/specificity (true negative rate) of those MATs were in the range of 80.0–100%/57.1–100% and 72.7–98.8%/82.2–100%, respectively.^15,16,22^

Transgenic cells constructed by using related signalling pathway molecules and reporter genes have been widely used to detect the bioactivity of biological products (cytokines (I type interferon, erythropoietin), hormones (GH, FSH and TSH), and monoclonal antibodies (anti-VEGF/VEGFR, anti-IL6/IL6R and anti-PD1/PDL1)).^42,43^ NF-κB is a central factor mediating inflammatory responses (described in the Introduction section). As shown in the study, we confirmed the feasibility of using NF-κB as a pyrogen marker to detect pyrogens. We also found a potential relationship between the activation of NF-κB and the secretion of proinflammatory cytokines and noted differences in the secretion of those cytokines (data not shown), which to some extent shows the representativeness and advantage of NF-κB as a pyrogenic marker.

An accurate understanding of product characteristics and technology is the premise for reasonably choosing pyrogen detection methods for controlling the safety of products. We found that there were few substances, including oxidised low-density lipoprotein, a poly (beta-amino ester), that could activate macrophages independent of NF-κB signalling.^44,45^ If a product contains other substances, a pyrogen detection method can be chosen or designed on a “case-by-case” basis depending on the raw materials, process and relevant mechanism for the product.

In summary, this study, from the establishment of the test in the laboratory to the validation of the test in different laboratories, systematically established a novel pyrogen test based on THP-1 cells transfected with a luciferase reporter gene regulated by NF-κB, which could provide a novel alternative for pyrogen detection.

## Materials and methods

### Reagents

THP-1 cells were obtained from ATCC. The reagents used included phosphate-buffered saline (PBS, Gibco, Shanghai, China, Cat# C10010500CP), Opti-MEM (Gibco, Shanghai, China, Cat# 31985070) and hygromycin B (Sigma‒Aldrich, Shanghai, China, Cat# V900372-1G). The national standard for bacterial endotoxins is LPS, which was obtained from *Escherichia coli* O55:B5 [9,000 endotoxin units (EU)/vial, batch 150800-201601, identical to the 2nd international WHO standard for endotoxin 94/580 from *E. coli* O113:H10] and was provided by the National Institutes for Food and Drug Control (NIFDC). Additional reagents included LPS (Sigma‒Aldrich, Shanghai, China, Cat# L2654-1MG, Cat# L6529-1MG, Cat# L4391-1MG, Cat# L4516-1MG, Cat# L2755-10MG, Cat# L5886-10MG, Cat# L7170-1MG, Cat# L4641-1MG, Cat# L6143-1MG, Cat# L7895-1MG, Cat# L4268-10MG), LTA (Sigma‒Aldrich, Shanghai, China, Cat# L3265-5MG), zymosan (Sigma‒Aldrich, Shanghai, China, Cat# Z4250-1G), peptidoglycan (Sigma‒Aldrich, Shanghai, China, Cat# 69554-10MG-F), lectin (Sigma‒Aldrich, Shanghai, China, Cat# L8754-5MG), β-1,3-glucan (Sigma‒Aldrich, Shanghai, China, Cat#89862-1G-F), RPMI 1640 (Gibco, Shanghai, China, Cat# C11875500BT), foetal bovine serum (FBS, Excell, Suzhou, China, Cat# FSP500), HEPES (Sigma‒Aldrich, Shanghai, China, Cat# H0887-100 ml), penicillin‒streptomycin (BasalMedia, Shanghai, China, Cat# S110JV), L-glutamine (Gibco, Shanghai, China, Cat# 25030081), bovine calf serum (BCS, Gibco, Shanghai, China, Cat# A3520502), mouse IgG (Jackson ImmunoResearch, West Grove, PA, USA, Cat# 015-000-003), human FcR blocking solution (Shanghai Maokang Biotechnology Co., Ltd., Shanghai, China, Cat# MX1505-50T), F(ab’)_2_ fragment goat anti-mouse IgG (Jackson ImmunoResearch, West Grove, PA, USA, Cat# 115-606-071), propidium iodide (Sigma‒Aldrich, Shanghai, China, Cat# P4170-10MG), paraformaldehyde (Biyuntian, Shanghai, China, Cat# P0099), Fixable Viability Stain reagent (BD Cat# 564997), Triton X-100 (Solarbio, Beijing, China, Cat# T8200), PE-mouse IgG1 (BioLegend, San Diego, CA, USA, Cat# 400114), PE-mouse anti-human CD14 (BioLegend, San Diego, CA, USA, Cat# 367104), Bright-Glo luciferase assay reagent (Promega, Madison, WI, USA, Cat# E2650), polymyxin B (Sigma‒Aldrich, Shanghai, China, Cat# P1004-10MU), pyrogen-free water for the BET (Zhanjiang A&C Biological Ltd., Zhanjiang, Guangxi, China), group A & C meningococcal polysaccharide vaccine (Yuxi Walvax Biotechnology Co. Ltd., Yuxi, China, batch No. D201805064), basiliximab (Novartis Pharma Stein AG, Beijing, China, batch No. SFT91), rabies vaccine (Vero cells) for human use, freeze-dried (Liaoning Chengda Biotechnology Co. Ltd., Shenyang, China, batch No. 201804084), Japanese encephalitis vaccine (Vero cells), inactivated (Liaoning Chengda Biotechnology Co. Ltd., Shenyang, China, batch No. 201709B16), insulin aspart injection, (Beijing Double-Crane Pharmaceutical Co. Ltd., batch No. 20161203), human albumin (Nanyue Biopharming Co. Ltd., China, batch No. 201805016) and recombinant human erythropoietin injection (CHO cell) (Harbin Pharmaceutical Group Biological Engineering Co. Ltd., Harbin, China, batch No. 20170404).

### Consumables

The consumables used included 96-well plates [Corning, NY, USA, Cat# 3917 (flat bottom, white polystyrene, tissue culture treated), Cat# 9018 (flat bottom clear, polystyrene, high binding surface)], 96-well plates (JET Biofil, Guangzhou, China, Cat# TCP002096, U-bottom clear), 0.20 µm syringe filters (Millipore, Shanghai, China, Cat# SLLGX13NL) and human IL-1β, IL-6 and TNF-α ELISA kits (R&D, Shanghai, China, Cat# DY201-5, Cat# DY206, Cat# DY210-05). Other reagents/materials were purchased as sterile and free of pyrogens, and the glassware was baked at 250 °C for 1 h.

Construction and transfection of the plasmid pCM1.1_luciferase (luc)_NFκB_hygromycin (hygro)

Overlap PCR was used to synthesise the NF-κB response element (5_-TCCTCGGAAAGTCCCCTCTGAGATCCTCGGAAAGTCCCCTCTGAGATCTCAGAGGGGACTTTCCGAGGA-3_), which was inserted into the multiple cloning site ahead of the minimal promoter region in the plasmid pCM1.1_luc_hygro and then DNA sequencing was used to identify the positive clone (named pCM1.1_luc_NFκB_hygro).

THP-1 cells (ATCC) were transfected with pCM1.1_NF-κB_luc_hygro by electroporation and then cultured in complete medium (RPMI 1640 supplemented with 10% FBS, 25 mM HEPES, 1% penicillin‒streptomycin and 2 mM L-glutamine) at 37 °C in an atmosphere of 5% CO_2_ aerobically for 48 h. Then, the cells were cultured in complete medium supplemented with 200 μg/ml hygromycin B for 2–3 months and the hygromycin-resistant cells were sorted by limited dilution to obtain monoclonal cells, which were screened for luciferase activity by treatment with LPS. The resulting positive clones (named THP-1_NF-κB_luc_cells) were routinely maintained in complete medium supplemented with 200 μg/ml hygromycin B.

### Procedure for the novel pyrogen test

THP-1_NF-κB_luc_cells in the logarithmic growth stage were collected by centrifugation (200 × *g* for 5 min), resuspended in the assay medium (RPMI 1640 supplemented with 1% FBS) at the needed concentration and seeded into 96-well plates (50 μl/well). Sample solutions prepared with the assay medium were added to 96-well plates (50 μl/well, *n* = 3 or 4). The plates were aerobically incubated at 37 °C in an atmosphere of 5% CO_2_ for the indicated period. After incubation, Bright-Glo luciferase assay reagent was added to 96-well plates (100 μl/well), which were subsequently shaken for 2 min. Finally, the fluorescence intensity was determined using a microplate reader (SpectraMax i3X) and expressed as relative light units (RLUs).

### FACS

For cell-surface staining, THP-1 cells were harvested by centrifugation (200 × *g* for 5 min), washed twice with ice-cold PBS, resuspended in PBS containing 4% BCS, 200 μg/ml mouse IgG and human FcR blocking solution at a concentration of 2 × 10^6^ cell/ml and aliquoted into 96-well plates (50 μl/well), which were placed on ice for 15 min. Then, the cells were stained with mouse anti-TLR1 (Invitrogen, Shanghai, China, Cat# 16-9911-82), TLR6 (BioLegend, San Diego, CA, USA, Cat# 334708), TLR10 (BioLegend, San Diego, CA, USA, Cat# 354604) and PE-mouse anti-human CD14 (BioLegend, San Diego, CA, USA, Cat# 367104), with PE-mouse IgG1 as an isotype control. Meanwhile, the cells were stained with mouse anti-TLR2 (R&D, Shanghai, China, Cat# FAB2616R), TLR4 (R&D, Shanghai, China, Cat# FAB6248A) and TLR5 (R&D, Shanghai, China, Cat# FAB6704R), and AF647-mouse IgG1 was used as an isotype control. The cell-antibody mixture was incubated on ice for 45 min in the dark, washed three times with PBS containing 4% BCS and resuspended in 200 μl of 5 μg/ml propidium iodide in PBS to stain dead cells. Data were collected using a BD FACS Celesta system and analysed with FlowJo software. For intracellular staining of TLRs [e.g., TLR3 (Invitrogen, Shanghai, China, Cat# 12-9039-82), TLR7 (R&D, Shanghai, China, Cat# IC5875P), TLR8 (R&D, Shanghai, China, Cat# IC8999R) and TLR9 (R&D, Shanghai, China, Cat# IC36582R)], the cells were stained with fixable viability stain reagent (BD, Cat# 564997), which is a kind of live/dead dye. Then, the washed cells were fixed in 4% paraformaldehyde for 15 min, washed three times with PBS containing 0.2% Triton and blocked; detection was performed as described above.

### Statistical analysis

All experiments were repeated three times. The data are expressed as the mean and standard deviation (SD) of the mean. The data were compared between groups using Student’s *t* test.

### Supplementary information

Figure S1a, Figure S1b

## Data Availability

All data and THP-1_NF-κB_luc_cell from the current study are available from the corresponding author upon reasonable request.

## Material availability

All data and THP-1_NF-κB_luc_cell from the current study are available from the corresponding author upon reasonable request.

## References

[1] Fennrich S (2016). More than 70 years of pyrogen detection: current state and future perspectives. Altern. Lab. Anim..

[2] Dullah EC, Ongkudon CM (2017). Current trends in endotoxin detection and analysis of endotoxin-protein interactions. Crit. Rev. Biotechnol..

[3] Franco E (2018). Endotoxins from a pharmacopoeial point of view. Toxins.

[4] Spoladore J (2021). Standardized pyrogen testing of medical products with the bacterial endotoxin test (BET) as a substitute for rabbit pyrogen testing (RPT): a scoping review. Toxicol. In Vitro.

[5] da Silva CC, Presgrave OA, Hartung T, de Moraes AM, Delgado IF (2016). Applicability of the monocyte activation test (MAT) for hyperimmune sera in the routine of the quality control laboratory: Comparison with the rabbit pyrogen test (RPT). Toxicol. In Vitro.

[6] Hartung T (2021). Pyrogen testing revisited on occasion of the 25th anniversary of the whole blood monocyte activation test. ALTEX.

[7] Hartung T (2015). The human whole blood pyrogen test—lessons learned in twenty years. ALTEX.

[8] Vipond C, Findlay L, Feavers I, Care R (2016). Limitations of the rabbit pyrogen test for assessing meningococcal OMV based vaccines. ALTEX.

[9] Valentini S (2019). Monocyte-activation test to reliably measure the pyrogenic content of a vaccine: an in vitro pyrogen test to overcome in vivo limitations. Vaccine.

[10] Tamura H, Reich J, Nagaoka I (2021). Outstanding contributions of LAL technology to pharmaceutical and medical science: review of methods, progress, challenges, and future perspectives in early detection and management of bacterial infections and invasive fungal diseases. Biomedicines.

[11] Bolden J (2020). Currently available recombinant alternatives to horseshoe crab blood lysates: are they comparable for the detection of environmental bacterial endotoxins? A review. PDA J. Pharm. Sci. Technol..

[12] Marius M, Vacher F, Bonnevay T (2020). Comparison of limulus amoebocyte lysate and recombinant factor c assays for endotoxin detection in four human vaccines with complex matrices. PDA J. Pharm. Sci. Technol..

[13] Marius M, Vacher F, Bonnevay T (2020). Comparison of bacterial endotoxin testing methods in purified pharmaceutical water matrices. Biologicals.

[14] Hartung T (2001). Novel pyrogen tests based on the human fever reaction. The report and recommendations of ECVAM workshop 43. European centre for the validation of alternative methods. Altern. Lab. Anim..

[15] Hoffmann S (2005). International validation of novel pyrogen tests based on human monocytoid cells. J. Immunol. Methods.

[16] Schindler S (2006). International validation of pyrogen tests based on cryopreserved human primary blood cells. J. Immunol. Methods.

[17] Stoppelkamp S (2017). Speeding up pyrogenicity testing: Identification of suitable cell components and readout parameters for an accelerated monocyte activation test (MAT). Drug Test Anal..

[18] Vipond C (2019). Development and validation of a monocyte activation test for the control/safety testing of an OMV-based meningococcal B vaccine. Vaccine.

[19] Molenaar-de Backer MWA, Gitz E, Dieker M, Doodeman P, Ten Brinke A (2021). Performance of monocyte activation test supplemented with human serum compared to fetal bovine serum. ALTEX.

[20] Hasiwa N (2013). Evidence for the detection of non-endotoxin pyrogens by the whole blood monocyte activation test. ALTEX.

[21] Stang K (2014). Highly sensitive pyrogen detection on medical devices by the monocyte activation test. J. Mater. Sci. Mater. Med..

[22] He Q (2018). Analysis of IL-6 and IL-1β release in cryopreserved pooled human whole blood stimulated with endotoxin. Innate Immun..

[23] Studholme L (2019). Evaluation of the monocyte activation test for the safety testing of meningococcal B vaccine Bexsero: a collaborative study. Vaccine.

[24] Nakagawa Y, Maeda H, Murai T (2002). Evaluation of the in vitro pyrogen test system based on proinflammatory cytokine release from human monocytes: comparison with a human whole blood culture test system and with the rabbit pyrogen test. Clin. Diagn. Lab. Immunol..

[25] Broom M (2007). Physiology of fever. Paediatr. Nurs..

[26] Muszynski A, Laus M, Kijne JW, Carlson RW (2011). Structures of the lipopolysaccharides from Rhizobium leguminosarum RBL5523 and its UDP-glucose dehydrogenase mutant (exo5). Glycobiology.

[27] Gnauck A, Lentle RG, Kruger MC (2016). The characteristics and function of bacterial lipopolysaccharides and their endotoxic potential in humans. Int. Rev. Immunol..

[28] Cot M (2011). Lipoteichoic acid in Streptomyces hygroscopicus: structural model and immunomodulatory activities. PloS one.

[29] Sahoo BR (2013). Elucidation of novel structural scaffold in rohu TLR2 and its binding site analysis with peptidoglycan, lipoteichoic acid and zymosan ligands, and downstream MyD88 adaptor protein. BioMed Res. Int..

[30] Walachowski S, Tabouret G, Foucras G (2016). Triggering dectin-1-pathway alone is not sufficient to induce cytokine production by murine macrophages. PloS one.

[31] Dillon S (2006). Yeast zymosan, a stimulus for TLR2 and dectin-1, induces regulatory antigen-presenting cells and immunological tolerance. J. Clin. Invest..

[32] Yu H, Lin L, Zhang Z, Zhang H, Hu H (2020). Targeting NF-κB pathway for the therapy of diseases: mechanism and clinical study. Signal Transduct. Target. Ther..

[33] He Q (2020). A novel reporter gene assay for pyrogen detection. Jpn. J. Infect. Dis..

[34] Borton LK, Coleman KP (2018). Material-mediated pyrogens in medical devices: applicability of the in vitro monocyte activation test. ALTEX.

[35] Tsuchiya S (1980). Establishment and characterization of a human acute monocytic leukemia cell line (THP-1). Int. J. Cancer.

[36] Chanput W, Mes JJ, Wichers HJ (2014). THP-1 cell line: an in vitro cell model for immune modulation approach. Int. Immunopharmacol..

[37] Pardo-Ruiz Z (2016). Soluble β-(1,3)-glucans enhance LPS-induced response in the monocyte activation test, but inhibit LPS-mediated febrile response in rabbits: Implications for pyrogenicity tests. Eur. J. Pharm. Sci..

[38] Lilley E (2023). Integrating 3Rs approaches in WHO guidelines for the batch release testing of biologicals: responses from a survey of vaccines and biological therapeutics manufacturers. Biologicals.

[39] Cirefice G (2023). The future of pyrogenicity testing: phasing out the rabbit pyrogen test. A meeting report. Biologicals.

[40] Gimenes I, Caldeira C, Presgrave OA, de Moura WC, Villas Boas MH (2015). Assessment of pyrogenic response of lipoteichoic acid by the monocyte activation test and the rabbit pyrogen test. Regul. Toxicol. Pharmacol..

[41] Han Q (2019). Application of a TLR overexpression cell model in pyrogen detection. Biotechnol. Bioeng..

[42] Lei Y, Yong Z, Junzhi W (2023). Development and application of potency assays based on genetically modified cells for biological products. J. Pharm. Biomed. Anal..

[43] Wang C (2022). Development and validation of a novel luciferase reporter gene assay to detect pyrogen. Biologicals.

[44] Dold NM, Zeng Q, Zeng X, Jewell CM (2018). A poly(beta-amino ester) activates macrophages independent of NF-κB signaling. Acta Biomater..

[45] Schwarz A, Bonaterra GA, Schwarzbach H, Kinscherf R (2017). Oxidized LDL-induced JAB1 influences NF-κB independent inflammatory signaling in human macrophages during foam cell formation. J. Biomed. Sci..

