# Supplementary material for: A novel alternative for pyrogen detection based on a transgenic cell line
Source: Signal Transduct Target Ther. 2024 Feb 19;9:33. doi: 10.1038/s41392-024-01744-0 (PMC10874988; doi:10.1038/s41392-024-01744-0)
Supplement: Supplementary file 1 — Figure S1a, Figure S1b [file 41392_2024_1744_MOESM1_ESM.pdf]

Supplementary Materials for

A novel alternative for pyrogen detection based on a transgenic cell line

Qing He, Chuan-Fei Yu, Gang Wu, Kai-Qin Wang, Yong-Bo Ni, Xiao Guo, Zhi-Hao Fu, Lan Wang, De-Jiang Tan, Hua Gao, Can Wang, Gang Chen, Xu-Hong Chen, Bo Chen, Jun-Zhi Wang

Correspondence to: wangjz@nifdc.org.cn

This PDF file includes:

Figure. S1

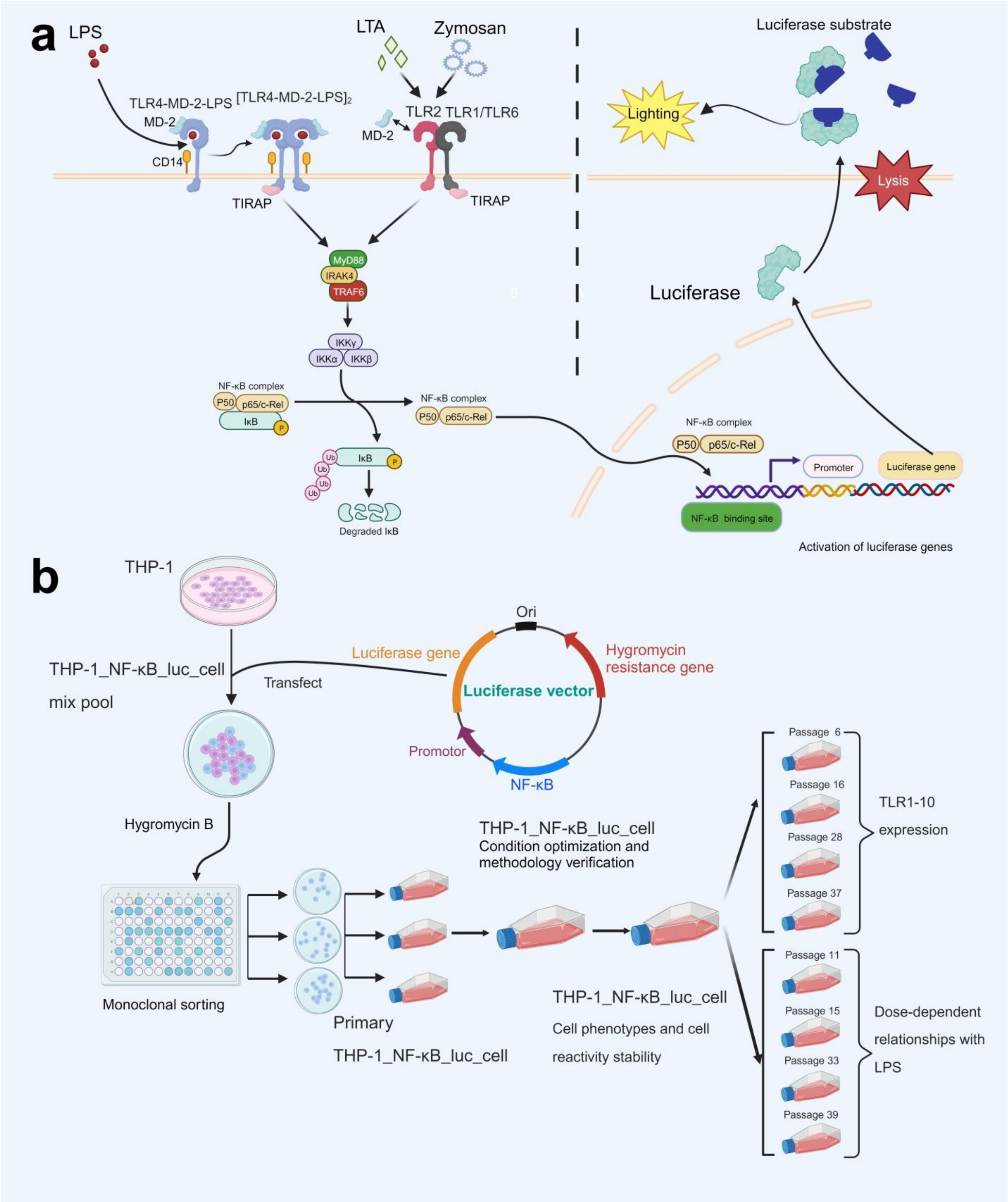

Figure. S1.

Principle and technical route of the test. **a** Mechanism of the test for detecting pyrogens. **b** Process of establishing a THP-1_NF-κB_luc_cell that is a subclone sensitive to endotoxin and evaluating the stability of the cell reactivity and cell phenotypes at different cell passages.
